# Weak evidence base for bee protective pesticide mitigation measures

**DOI:** 10.1093/jee/toad118

**Published:** 2023-07-17

**Authors:** Edward A Straw, Dara A Stanley

**Affiliations:** School of Agriculture and Food Science, University College Dublin, Dublin, Ireland; School of Agriculture and Food Science, University College Dublin, Dublin, Ireland

**Keywords:** bee, pesticide, systematic review, mitigation measure, mitigation, repellent

## Abstract

Pesticides help produce food for humanity’s growing population, yet they have negative impacts on the environment. Limiting these impacts, while maintaining food supply, is a crucial challenge for modern agriculture. Mitigation measures are actions taken by pesticide users, which modify the risk of the application to nontarget organisms, such as bees. Through these, the impacts of pesticides can be reduced, with minimal impacts on the efficacy of the pesticide. Here we collate the scientific evidence behind mitigation measures designed to reduce pesticide impacts on bees using a systematic review methodology. We included all publications which tested the effects of any pesticide mitigation measure (using a very loose definition) on bees, at any scale (from individual through to population level), so long as they presented evidence on the efficacy of the measure. We found 34 publications with direct evidence on the topic, covering a range of available mitigation measures. No currently used mitigation measures were thoroughly tested, and some entirely lacked empirical support, showing a weak evidence base for current recommendations and policy. We found mitigation measure research predominantly focuses on managed bees, potentially failing to protect wild bees. We also found that label-recommended mitigation measures, which are the mitigation measures most often applied, specifically are seldom tested empirically. Ultimately, we recommend that more, and stronger, scientific evidence is required to justify existing mitigation measures to help reduce the impacts of pesticides on bees while maintaining crop protection.

## Introduction

The majority of modern agriculture relies on pesticides to protect yields (Willer et al. 2021). So, while pesticides can have negative environmental impacts (Goulson 2013, McArt et al. 2017, Cullen et al. 2019), they are currently an integral part of ensuring sufficient global food production (Crowder and Illan 2021). While much attention surrounding the negative environmental impacts of pesticides focuses on removing substances from use (Goulson 2018, PAN 2023), progress has been limited largely due to a lack of alternative options available to maintain production (Beckie et al. 2020, but see Jactel et al. 2019). It is likely a variety of methods will be needed to reduce the environmental damage of pesticides (Beckie et al. 2020), including options to mitigate their impacts where they continue to be used.

Pesticides can cause harm to the environment in many ways, including threatening bee populations (Potts et al. 2016). Bees pollinate many agricultural crops (Klein et al. 2007), and as pollinators, they are critical members of the wider ecosystem (Ollerton et al. 2011, Rodger et al. 2021). Many bee species are in decline, including 7.7% of European bee species, while a further 79% of species have unknown population trends (Nieto et al. 2014). This image is mirrored globally, with even higher proportions of data-deficient species (Potts et al. 2016). Numerous stressors are involved in the declines, with changes in land use particularly important (Hemberger et al. 2021). There is, however, rigorous evidence that some pesticides, as used in the field, have negative consequences for bees (Rundlöf et al. 2015, Woodcock et al. 2016, McArt et al. 2017, Motta et al. 2018, Tamburini et al. 2021). So, reducing this harm to the maximal extent should be the upmost priority.

Many governments are making considerable efforts to shift away from widespread pesticide usage, through guidance, incentives, and pressure (Liu et al. 2005, European Commission 2009, 2021a, Environmental Protection Agency 2021). One key example here is the European Union Farm to Fork strategy which aims to reduce pesticide use and the risks of pesticides by 50% by 2030 (European Commission 2021b). As pesticides are part of our food production system, and will be for the foreseeable future, it is appropriate to ask not just how can we reduce pesticide usage, but also, how can we reduce the impacts of pesticide use?

How pesticides are used is critical in determining their environmental toxicity. For example, an insecticide applied to a tree in flower could be damaging to bees and other pollinators (Hatfield et al. 2021), while application out of bloom may cause little to no harm (Havstad et al. 2019). So, it is theoretically possible that the environmental impact of pesticides can be at least partially uncoupled from their usage through the implementation of “mitigation measures.” Mitigation measures are actions taken which reduce the potential negative effects of a pesticide application, while still allowing the application. These include actions such as using low-drift nozzles or only applying pesticides in certain weather conditions.

Many mitigation measures are already mandated on the pesticide product label or in governmental guidance documents (OECD 2006). Within the European Union at least, following these measures is a legal requirement, with penalties for noncompliance (European Commission 2008, 2009). Farmers within the European Union also report high levels of compliance with pesticide regulations pertinent to protecting bee health (Straw et al. 2023). In many cases, measures may be suggested as part of advice or guidance given to farmers in addition to what is written on label guidelines. A few notable measures include optionally applying pesticides outside of pollinator foraging activity hours, and taking practical measures to protect managed bees such as covering colonies during application (Moffett et al. 1977, Moffet et al. 1979, 1981).

Given the scale and impact of pesticide usage, it is important that mitigation measures used are effective (Randall et al. 2015). While most mitigation measures designed to protect bees are practical common-sense ideas, we appraised the scientific evidence behind mitigation measures using a systematic review. Below we discuss the knowledge compiled, what it tells us about existing measures, what measures could be adopted additionally, and, crucially, what gaps there are in our current understanding.

## Methods

We searched the literature for any publications presenting evidence on the efficacy of any pesticide mitigation measure in reducing the impacts of the pesticide on bees. We define a mitigation measure very broadly as “actions taken which reduce the potential negative effects of a pesticide application, while still allowing the application.” We define an impact as any measurable effect on bees covering all scales from individual bees through to the population level. A Web of Science Core Collection search was undertaken in October 2021 using the PRISMA framework (Moher et al. 2009). An initial search was undertaken with broadly defined search terms on the theme of pesticide mitigation measures, including a term for mitigation AND a term for bees AND a term for pesticides. This was followed by a series of supplementary searches for specific mitigation measures. In total 4,161 publications were found, with 2,957 unique publications (see Supplementary Figs. S1 and S2 and Supplementary Section S2 for more details).

The following terms were used in a Topic, Title, and Abstract Search for the main search:

((mitigation OR mitigat* OR reduce OR reduction OR reduc* OR exposure OR protect OR avoid OR alleviat* OR loss* OR ameliorat* OR conserv*)

**AND**

(honeybee OR bumblebee OR bee OR bees)

**AND**

(pesticid* OR herbicid* OR fungicid* OR adjuvant OR co-formulant OR coformulant OR insecticide OR molluscicide OR (plant protection product) OR agrochem* OR (agro-chem*) OR agrichem* OR (agri-chem*)))

After screening the results of this high-yield search and identifying the range of mitigation measures tested in the literature, a supplementary search protocol was used. Here 16 narrow, low yielding, searches were used to supplement the main search. Not all mitigation measures could be included in a supplementary search as the terms to parameterize them were too vague, that is, crop type. Narrow searches were based on mitigation measure identified from the results of the initial broad search alongside other search terms based on the FAO Pesticide Registration Toolkit (Food and Agriculture Organisation 2022) and published academic literature, to ensure a broad base of literature was captured. The supplementary searches (December 2021 through April 2022) retained the bee and pesticide terms, while swapping the broad mitigation term for a specific mitigation measure term (see Supplementary Section S1 for full terms).

Our primary inclusion criteria were that a peer-reviewed publication presents a study(ies) where a measure is taken to reduce the impact of a pesticide application on bees, and the efficacy of this is recorded. We define what a measure taken could be very broadly and included measures of ambiguous relevance. We define the efficacy of the measure as any measurable impact on the bees tested (individual, colony, or population level). Beyond this broad definition of a measured impact, we also include any publications that measure a change in pesticide residues in a matrix that has been collected by bees (honey, wax, bee bread, pollen, nectar, and water; see Supplementary Section S1). An example of a relevant publication would be one which tested whether applying a pesticide at night (the mitigation measure) impacted bee populations (level of impact on bees, and the measured impact on bees).

Data were categorized for the sake of visual clarity in the graphs and broad overview statistics. These categorisations were not definitive and are only for thematic interpretation. One rationale for this is that research into mitigation measures is a disparate noncodified field, with few publications. The Publications by Topic section under Results discusses each paper individually, and this is a truer representation of the diversity of topics. This section demonstrates the diversity of measures taken. We chose not to perform a meta-analysis on the dataset as the research found was too disparate (both in its methods and the topics covered) to facilitate a quantitative comparison. The Publications by Topic section lays out the key findings such that they may be judged qualitatively. Some publications test multiple species, substances or topics, and thus can be double counted in the figures and text. The comparisons of research quantity presented in the figure legends used chi-squared goodness-of-fit tests, all assumptions were met.

Other evidence sources do exist, which provide information on the efficacy of mitigation measures, without directly experimentally testing them. We acknowledge that their exclusion underestimates the level of knowledge we have underpinning mitigation measures. Evidence from regulatory testing, both toxicity and residue level, and evidence submitted to regulatory bodies may not be captured in this review if not published through a peer-reviewed scientific journal.

## Results

**Fig. 1.**
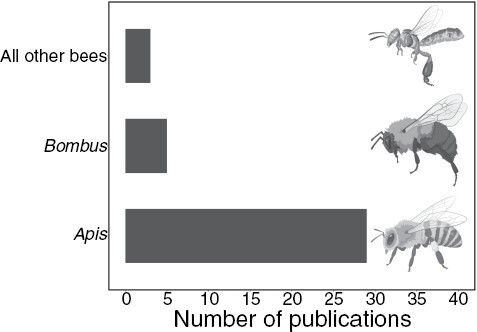
The number of publications on pesticide use mitigation measures relating to each bee group. Honey bees are the most researched taxa, with significantly more publications (χ^2^ =31.324, *P* < 0.0001). Note some publications tested multiple taxa. Illustrations from BioRender.com.

Thirty-three publications were found that met our criteria, with one further publication identified from our knowledge of the literature. Of these publications, 14 were from the United States, with less than 5 coming from every other nation represented. Twenty-nine publications tested on honey bees, while just 5 tested on bumble bees, and just 3 on all other bee species (see Fig. 1). Among honey bees, *Apis mellifera* was most tested, although 4 publications tested *Apis florea*.

**Fig. 2.**
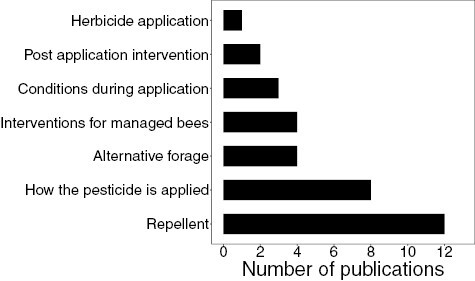
The number of publications in each category of mitigation measures identified. Niche research questions have been aggregated into broader categories (i.e. how the pesticide is applied, which includes nozzle selection and the comparison of seed treatment versus spraying) for the sake of presentation.

The most common mitigation measure tested was repellents designed to repel bees from visiting recently treated crops, with 12 publications. Eight tested changes to how the pesticide is applied, with 2 testing things that can be done after the pesticide is applied (both irrigation). Four tested alternative forage (food and water) and another 4 tested interventions for managed bees. Three tested changes to the conditions when the pesticide is applied (season and humidity), and finally one tested the application of a herbicide to prevent exposure to other pesticides (see Fig. 2).

Just 10 publications measured residue data in relation to the mitigation measure. Where publications mimicked pesticide application, how they mimicked it varied considerably. Most used a spray (*n* = 16), while 7 others used a spiked food source. Seven publications used seed treatment dust, 3 used cubes of agarose gel, 1 used a soil drench, and another used spiked irrigation liquid. Nearly all publications were agriculturally focused (*n* = 26), with just one from horticulture, while 7 publications did not specify their focus.

None of the publications on repellents used a pesticide alongside the repellent, but most other publications did use a pesticide to test their mitigation measure. Due to the long timespan covered by the literature, few active substances are repeatedly tested with the exception of some neonicotinoids.

Insecticides were the most common active ingredients studied (*n* = 41), and there were just 4 fungicides, 1 herbicide, and 3 combination exposures. Not all experiments applied substances to a crop, but those that did were very varied. Seven publications used corn, while cotton and oilseed rape were next, both with 3 publications each, as well as several other crops.

### Publications by Topic

#### How the pesticide is applied

How a pesticide is applied is important in defining its toxicity to bees, and 8 publications investigated this measure. Two publications have tested how spray parameters can impact bees, with Cabrera-Marín et al. (2016) finding finer sprays less harmful to bees, while Perine et al. (2021) found that nozzle choice can impact active ingredient deposition onto bees. A new design of sprayer configuration, which sprays beneath the canopy, has been tested giving inconclusive results (Trodtfeld et al. 2017).

Pesticides are not all sprayed, and seed treatments are also commonly used. However, as treated seeds are drilled, they can kick off toxic dust (Julius-Kuhn Institute 2008). Sgolastra et al. (2012) found this dust to be equally toxic gram for gram as spray deposits. Georgiadis et al. (2012) found that batches of seed that produce less dust may be safer for bees, although the treatments were not perfectly comparable. To reduce the issue of dust, Girolami et al. (2013) and Pochi et al. (2015) develop new types of drilling machine exhausts, with the former unsuccessful but the latter modeled to have a ~85% reduced deposition on flying bees.

#### Buffer zone

Agri-environment schemes often promote buffer zones around fields, to provide space for nature, and mitigate spray drift. Davis and Williams (1990) found that different pesticides dissipate in the environment very variably based on climatic conditions. Buffer zones of 4–40 m were found necessary to avoid exposing bees to dangerous levels of pesticides, with the distance required both pesticide and environmental condition dependent.

#### Weather conditions during application

**Fig. 3.**
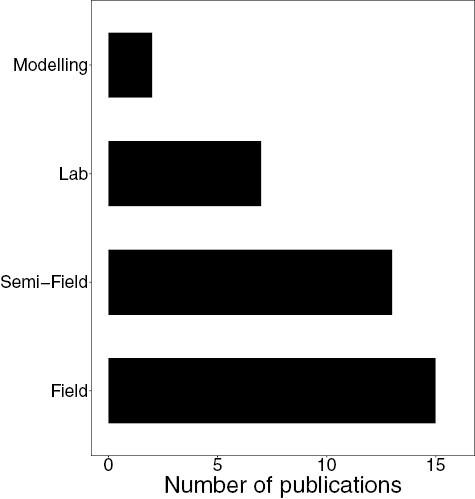
The number of publications that involved each experimental design category. Field studies are the most prevalent, with a statistically significant difference in the number of studies in each category (χ^2^ = 10.351, *P* = 0.0158).

Three publications tested the impacts of humidity on pesticide toxicity after bees are exposed to seed treatment dust. Marzaro et al. (2011) and Girolami et al. (2011, 2013) all found insecticide dust more lethal at high versus low humidity. Using published data, Davis and Williams (1990) modeled bee exposure to various pesticides in different weather conditions, finding highly stable air conditions exacerbated spray drift and deposition onto bees (modeling papers were the least common approach, see Fig. 3).

#### Crop stage

An existing mitigation measure applied to many insecticides is restrictions on crop stage during application. Typically, this is a restriction on spraying a pesticide during the flowering stage of the crop, or shortly before. Just one publication has tested this though, with Havstad et al. (2019) finding that application of a neonicotinoid insecticide during clover flowering led to much higher detections in bumblebees than when the same application was made before flowering.

#### Irrigation postapplication

Irrigation is used to keep crops well-watered, but it can also move pesticide residues down into the soil. Just 2 publications have tested this in relation to pesticide impacts on bees. Gels et al. (2002) found that irrigation of red clover after a neonicotinoid application wholly mitigated the reproductive impacts on *Bombus impatiens*, while Cecala et al. (2021) found that irrigation of ornamental plants did not benefit *Megachile rotundata* reproduction or foraging activity (although methods used may not allow direct detection of an effect).

#### Using herbicides to remove flowering weeds before pesticide application

Insecticide labels often recommend removing flowering weeds from the area to be sprayed (e.g., Closer Label, Syngenta 2021) to prevent floral resources being contaminated and avoid direct overspray of bees while they forage (Straw et al. 2022) One option is to use a herbicide to remove the flowering weeds, and one publication has tested this. McDougall et al. (2021) found that using a herbicide around an orchard significantly reduced bee numbers at both the ground and tree level.

#### Providing bees with alternative forage to treated crops

Many landscapes are dominated by agriculture, and such monocultural landscapes can lead to more limited diets for pollinators. Two publications tested the impacts of supplemental forage for reducing the impacts of a pesticide application on bees. Ingwell et al. (2021) found that supplemental forage (clover) mitigated the impacts of a neonicotinoid application to watermelon crops on *Bombus terrestris* colonies. Okubo et al. (2021) found that higher visitation to a supplemental patch of mustard reduced deaths from insecticide treatment of nearby rice paddies (however the experimental methods do not allow attribution of the effect to the supplemental forage alone).

#### Protecting managed bees through direct interventions

Interventions can be made when managed bees are set to be exposed to pesticides, with the aim of directly mitigating the impacts. The extreme example of this is the use of medicinal compounds that directly counteract the pesticide itself. Chen et al. (2021) tested pollen-inspired enzymatic microparticles, which counteract organophosphate insecticides. In laboratory conditions, they report that a 100% lethal dose of insecticide can be mitigated to 0% mortality in *Bombus impatiens*.

A less extreme intervention to protect managed bees is providing them with a clean water source after a pesticide application, to reduce their exposure to the pesticide through contaminated drinking water, and 5 publications tested this. McCune et al. (2021) provided managed honeybees water feeders, which were replenished after pesticide application. They found considerably lower agrochemical residue levels in their water feeders, versus natural water sources, and benefits for the survival of the bees. Rosa-Fontana et al. (2020) found that the stingless bee, *Melipona scutellaris*, had lower mortality when exposed to a neonicotinoid insecticide if they also had an uncontaminated water source. A series of publications found that honeybees supplied with supplemental water had higher survival when exposed to pesticide applications in nearby fields (Moffet et al. 1977, Moffet et al. 1979, 1981).

### Direct Interventions Prior to Pesticide Application

In a series of experiments in the United States, the impacts of physical intervention by a beekeeper prior to a pesticide application were tested (Moffet et al. 1977, Moffet et al. 1979, 1981). They trialed: shading the hives, covering the hives in burlap, a dead bee trap on the hive entrance, changes to the hive dimensions, supplemental pollen, syrup or water, confining the colonies to exclusion cages, moving the colonies to another site for a variety of timespans, and several treatments with multiple interventions. The provisioning of supplementary resources alone was reasonably effective, but the highest success came with combinations of interventions. Of particular effectiveness was a mixture of measures to prevent worker exposure, i.e. limiting their access out of the hive, paired with supplemental resources to limit the consequence of the loss of foraging opportunity (Moffet et al. 1977, Moffet et al. 1979, 1981).

### Repellents

To capitalize on bee’s sensory abilities (Beekman and Ratnieks 2001, Dyer 2012), repellent additives to pesticide sprays have been proposed. Repellents would dissuade bees from visiting a sprayed crop, limiting their exposure to pesticides. There are 12 publications on this topic, although all focus on honeybees.

Solomon and Hooker (1989), Mishra and Sihag (2009a), and Naik et al. (2010) tested a range of chemicals using a feeding assay methodology. They found high levels of repellence for some chemicals, even at low concentrations (0.3%). Mishra and Sihag (2010) had 2 subsequent publications, finding fixative compounds (ethylene glycol and glycerol), led to a more persistent repellent effect. Mishra and Sihag (2009b) found that the most successful chemicals from the semifield work also showed repellence of up to 100% immediately after application, dropping to 80% at 3 h.

A network of authors from the United States developed a laboratory-based protocol for screening substances for repellence using video tracking (Larson and Anderson 2017) and then corroborated this methodology with electroantennogram recordings (Larson et al. 2020), ultimately applying the results of the laboratory work in a feeding assay test and 2 semifield tests. In both flowering knapweed and watermelon crops, the application of piperidine was effective in reducing bee visitation 3 min after application (Larson et al. 2021).

Atkins et al. (1975) tested 12 chemicals for repellence. Several chemicals were rejected from testing for causing phytotoxicity. Some chemicals caused 70% repellence for 5.5 h. Free et al. (1985) found honeybee alarm pheromones to be effective, but short-lived repellents.

Mayer (1997) found methyl salicylate to be an ineffective repellent. In contrast, methyl salicylate and 2 other chemicals were found to be effective repellents by Sahebzageh et al. (2009), with fixative compounds improving the persistence.

## Discussion

We find evidence for several different mitigation measures available to farmers, beekeepers, and industry for reducing the exposure of bees to pesticides. Some measures, such as techniques to shield honeybee hives from pesticide exposure, are supported by a solid base of knowledge and could be adopted more widely (Moffet et al. 1977, Moffet et al. 1979, 1981). However, measures to protect wild, unmanaged bees are poorly developed, although promising options exist. A common key knowledge gap across measures is how mitigation measures would impact bees other than honeybees.

Pesticide labels contain several measures either specifically to protect bees (see Table 1) or generally to offer a reduction in environmental contamination. A surprising outcome of the review was that most research was on mitigation measures that the authors have not seen as label requirements (repellents, alternative forage, and interventions for managed bees). In fact, just 3 publications explicitly tested measures, which are on pesticide labels: (a) the timing of the application relative to the crop stage (Havstad et al. 2019), (b) herbicide application prior to insecticide application (McDougall et al. 2021), and (c) buffer zones (Davis and Williams 1990). This suggests a key knowledge gap, whereby the measures currently required of farmers are poorly substantiated by direct evidence in the scientific literature. As such, more research directly testing on-label mitigation measures is clearly justified.

**Table 1.** Mitigation measures that could protect bees and the number of publications testing them found in the systematic review

Measure	Note	Publications testing this measure
Restrict application to certain times of day	May prevent bee exposure to direct overspray	0
Restrict application to certain times of year	Could limit exposure of bees at sensitive times of year (early spring)	0
Restrict application to certain crop stages	Some pesticides have requirement not to treat crop during flowering which may reduce bee exposure	1
Restrict what crops can be treated	May prevent bee exposure by limiting application to bee-attractive crops	0
Limit number of times a pesticide can be used per season/year	May help reduce bee exposure by generally limiting pesticide use	0
Limit application rate of pesticide	May reduce level of exposure bees face	Not searched for
Restrict application methods	May have consequences for how bees are exposed to the pesticide	1
Require buffer zones to natural features	May reduce spray drift onto bees foraging around margins of field	1
Weather restrictions for application	May limit bee exposure or consequences of exposure	3
Remove flowering weeds	Designed to prevent insecticide residues on flowering weeds being an exposure route for bees	1
Restrictions on spray quality/parameters	May reduce bee exposure and alter the consequence of exposure	2
Drift reduction technology	Potentially reduces bee exposure through lower drift	0
Restrictions on volume of treatment solution	May alter drift or consequence of exposure to bees	0
Irrigation after a spray	May move pesticide residues away from flowers, reducing bees’ exposure	2
Include a repellent chemical in the pesticide spray	Could deter bee visitation to recently sprayed crops, reducing exposure	12
Interventions for managed pollinators	Could prevent exposure to and/or ameliorate or counteract the impacts of the pesticide	4
Providing alternative forage (floral or water) for pollinators	May ameliorate the impacts of pesticide exposure	3
Innovation to reduce environmental contamination from pesticide application	May lead to less pesticide residues in matrices bees visit, reducing their exposure	7

Several measures, which heavily impact the efficacy of a pesticide application, have been tested, notably the coarseness of a spray application (droplet size) and irrigation after application. However, a label recommendation or stipulation to modify either of these 2 parameters to protect bees would be confusing to farmers as it may contradict recommendations from equipment manufacturers on how to achieve an effective spray.

In a similar vein, while high humidity was found to cause additional toxicity from seed treatment dust (Girolami et al. 2011, 2013, Marzaro et al. 2011), it would not be prudent to stipulate that drilling can only occur at low humidity as it could heavily constrain the timing window in which drilling can occur. Conversely, air conditions that lead to high drift will cause both higher bee exposure in nearby areas and reduced deposition of the pesticide on the crop (Davis and Williams 1990). So here, the bee protective mitigation measure of spraying in optimal conditions is aligned with spray efficacy.

Several technological solutions to reduce environmental impacts of pesticides have been developed i.e. beneath canopy sprayers (Trodtfeld et al. 2017), dust-reducing exhaust designs (Girolami et al. 2013, Pochi et al. 2015) and nozzle technologies (Perine et al. 2021). Evidence for benefits of these novel technologies is sparse, but it is encouraging to see emerging research. Pursuing these technological solutions should be seen as a priority, as they offer an escape from the standard trade-off of bee protection versus yield protection.

Conversely, while some measures were found to be effective, their implementation may have wider environmental consequences that do not justify their use. One of these is herbicide application as a means to reduce bees insecticide exposure (McDougall et al. 2021). Herbicide application would remove forage from agricultural landscapes, which can already be forage poor, and expose bees directly to herbicides, which are not toxicologically benign (Straw et al. 2021). Nonchemical alternatives like mechanical mowing may be more ecologically friendly and have been found to reduce floral pesticide residues by as much as 99% (Larson et al. 2014).

Another mitigation measure of contentious value is medication for managed bee species to reduce the impacts of insecticide exposure (Chen et al. 2021). This potentially represents a step too far in the explicit protection of just-managed species. In the hypothetical scenario where a medicine mitigates 100% of the harm of a pesticide application in the managed species, this leads to the managed species being unaffected by the pesticide, while all other species are affected. This would heavily favor managed species in competition with wild species.

Alternative forage was tested in 2 publications on alternative food forage and 2 on alternative water forage. The promotion of supplemental forage for pollinators and wildlife in general is a well-supported notion for biodiversity conservation (Rundlöf et al. 2022), and one that is explicitly encouraged in policy and agri-environment schemes. Here we only find evidence in the literature for reducing pesticidal impacts from a single semifield experiment (Ingwell et al. 2021). So, while creating more diverse forage would benefit bees and nature generally, the evidence that it will do so by mitigating pesticide impacts is only emergent (but see Wintermantel et al. 2022). Providing alternative water forage for all bees is an evidence-supported measure farmers could pursue, albeit a relatively high effort one given the need to replenish them with fresh water after each individual spray. The benefits for non-*Apis* bees are also only assumed and may not be relevant for many species that do not collect water.

Dust from seed treatments was an exposure route in many publications. No reasonable measure to reduce its impact on bees was supported by multiple publications, although one seed drill design was reported to be promising (Pochi et al. 2015). The most promising route to ameliorate impacts appears to be legal limits on the amount of dust a batch of seeds can produce (Heubach-value limits) (Julius-Kuhn Institute 2008). While more testing is needed to determine whether low-dust-producing seed treatment batches are effective in protecting bees, it may be assumed. This would be effective in cutting off the source of the dust without substantively impacting drilling or efficacy.

One topic that dominated the literature (12/34 publications) was the use of repellent additives to pesticide sprays, with the potential to be effective in protecting wild and managed bees. Several promising chemicals were found as well as several methodologies for developing and validating their efficacy. The academic evidence is now limited by 2 factors, full-scale field trials and formulating constraints, which could also be addressed by the industry. Formulations can be constrained by difficulties mixing different substances, but as a range of chemicals have been found effective repellents it is unlikely none will be compatible with modern formulations.

Debate exists over how long the repellence would need to be. Solomon and Hooker (1989) argue that repellents would only need to deter visitation immediately after application, to prevent exposure to the worst of the chemical, while Atkins et al. (1975) suggest 12 h as the minimum effective period. Strong (>70%) repellence has been found in semifield trials up to 3 h, a promising length of time. It is not known if the successes from the semi-trials and small-scale field trials would map onto a real-world application though and whether bees may learn to ignore the repellence over time. Unfortunately, all research on repellents has thus far been conducted on honeybees, and given the considerable diversity of bees (Michener 2000), some species may respond differently.

In general, mitigation measures would benefit from an explicit understanding of what exposure to pesticides they are trying to avoid, direct overspray or longer-term residues. Residues of pesticides can remain in the nectar and pollen of crops, and the environment more broadly, for long time periods after application and pose a risk to bees in addition to direct spray (Cowles and Eitzer 2017). Measures like closing a honeybee colony for a day will largely remove the risk of direct overspray (with the bees confined to the hive), but once the hive is opened, they will be exposed to residues on crop flowers before they degrade entirely. What level of exposure is required to cause a meaningful impact on a bee is both pesticide specific and not well understood.

A notable omission from the literature is whether restrictions to the time of day a pesticide can be applied are effective. This is a common label restriction for many insecticides toxic to bees, with label language such as “If crop is to be sprayed during flowering, then spray in the evening or on a cloudy day when bees are not active in the crop” (e.g. Sparta Label, Corteva 2021) although many versions of this sentiment exist. No publications were found that quantified if this was an effective measure to take, meaning its widespread usage is not evidence based. While it is common sense that fewer bees will be active in the evening than in the middle of the day (Boyle-Makowski and Philogène 1985), when the evening starts is ambiguous, and unclear how it maps onto when bee foraging ends. Furthermore, a different assemblage of pollinators will be active at night. Work is needed to validate if this measure is actually effective in reducing the impacts of the pesticide on bees.

Overall, research has been far too heavily focused on honeybees, with just 7 of 34 publications testing non-*Apis* bees (which is a regular occurrence for bee research; Lundin et al. 2015, Cullen et al. 2019, Straw et al. 2022; see Figure 1). While an important species, the interests of honeybees are not always in line with all bee species and at times are even conflicting (Elbgami et al. 2014, Mallinger et al. 2017, Alger et al. 2019, Iwasaki and Hogendoorn 2022). The honey bee focus is most pertinently an issue in that over half the mitigation measures tested can only be applied to managed species (e.g., medicine, moving nests, supplemental syrup/pollen; Colla 2022). The development of these measures is understandable as beekeepers want to protect their bees from pesticides. However, application of these measures, if successful, would ameliorate the impacts of pesticides on the only group of bees with a substantive set of stakeholders (consumers, beekeepers, agricultural sector; Iwasaki and Hogendoorn 2021, 2022) and risks advocacy groups and policy settling for pesticide usage practices, which are not harmful to managed bees but are harmful to wild bees. This does not mean that it is unhelpful to research mitigation measures to specifically protect managed bees, merely that this needs to be accompanied by an equally fervent pursuit of techniques to protect wild bees. There is a growing body of evidence that honeybees can be damaging to wild bee populations (Elbgami et al. 2014, Iwasaki and Hogendoorn 2022), principally by the competition for resources and the transmission of diseases like deformed wing virus (Alger et al. 2019). Accordingly, measures that only protect honeybees may not be neutral toward other bees, but instead actively harmful to them.

Just as honeybees do not represent all bees, bees do not represent all pollinators. Other insects pollinate (Rader et al. 2015), as do a wide array of taxa, and other beneficial species exist. Hopefully, testing a more diverse range of bee species will help represent other species better.

Beyond a honeybee focus, there is also an explicit focus on insecticides (38 of 44 active ingredients tested), with little or no focus on other pesticide groups (Lundin et al. 2015, Cullen et al. 2019, Straw et al. 2022). However, bees may also be exposed to other pesticide classes, and this needs to be considered as other pesticide classes can be harmful to bees (Pilling and Jepson 1993, Cullen et al. 2019, Straw and Brown 2021).

To conclude, the evidence for currently used mitigation measures is sparse with several major knowledge gaps. Of prospective mitigation measures, many were reported to be effective, albeit with a small evidence base. Mitigation measures are but one tool that can be used to reduce the effects of pesticides on bees, and others should be explored also. In addition, whether mitigation measures target risks of over spray and/or residues needs further consideration. Far too much of a focus has been on measures to protect managed bee species specifically or using managed bee species to test measures, which would benefit all bees. For honey bees, a range of effective well-supported measures exist, such as direct interventions to shield honey bee hives from pesticide exposure. Repellent additives are a promising line of research, ready to be picked up by industry. Technological solutions like novel sprayer designs and seed treatment dust prevention are being trialed, with only limited success, but should be pursued further. While mitigation measures may not eliminate environmental harm, they have the potential to reduce it until farmers can be supported in moving away from pesticide-reliant agriculture. Ultimately, research on mitigation measures is a field ripe for further research which could substantively reduce how dangerous pesticides are for bees without serious consequences on pesticide efficacy.

## Supplementary Material

toad118_suppl_Supplementary_Material

## Data Availability

The authors intend to archive the data with DataDryad.
