# Supplementary material for: Weak evidence base for bee protective pesticide mitigation measures
Source: J Econ Entomol. 2023 Jul 17;116(5):1604–12. doi: 10.1093/jee/toad118 (PMC10564266; doi:10.1093/jee/toad118)
Supplement: toad118_suppl_Supplementary_Material [file toad118_suppl_supplementary_material.docx]

Supplementary Information

**Weak evidence base for bee protective pesticide mitigation measures.**

Edward A. Straw^1*^ and Dara A. Stanley^1^

^1^﻿School of Agriculture and Food Science, University College Dublin, Dublin, Ireland

*Corresponding author: EdwardAStraw@gmail.com

**Section S1: Supplementary Methods**

A series of loosely defined mitigation measures identified in the general pesticide literature and using the FAO Pesticide Registration Toolkit (FAO, 2022) were searched for using supplementary searches. To search for these, the following terms replaced the generic mitigation term in the supplementary searches, using the same broad pesticide and bee terms as the main search. Our search included only English language terms, and we only screened English language papers.

Supplementary Table 1. The supplementary search terms used and what they are parameterised based off. This does not directly relate to the categorisation of mitigation measures in Table 1, or the Results as these searches are simple attempts to capture as much relevant literature as possible.

| **Description of mitigation measure searched for** | **Search term used** |
| --- | --- |
| Use of alternative forage | Alternative Forage OR Alternative Resources |
| Provisioning of supplementary water | Waterer OR Water Feeder |
| Using irrigation to move residues off of crops into the soil | Irrigation OR Sub-Irrigation |
| Below canopy sprayers (called a dropleg sprayer) | Dropleg |
| Use of repellent additives | Repellent |
| Changing the timing of the application relative to the day, sun, crop stage or year | Dusk OR (Crop Stage) OR Evening OR Night OR Time of day OR Hour OR BBCH* OR Month OR Season |
| Covering managed bees in any which way to limit foraging during exposure | Covering |
| Changes to how the pesticide physically sprayed from | Nozzle OR Droplet OR Engineering Controls |
| Use of buffer strips around field edges | Buffer Zone OR Buffer Strip OR No Spray Strip OR No-Spray Strip |
| Changes to what height from which the pesticide is sprayed | Spray Height OR Boom Height |
| The type of formulation used | Formulation Type |
| Control of flowering weeds | Flowering Weeds |
| Use of aerial application | Aerial |
| Any method used to limit seed treatment excess dust | Dust |
| Changes to the volume of water used in an application | Water Volume OR Application Volume |
| Use of mowing to remove flowering weeds | Mow OR Mowing |
| When to spray relative to weather conditions | weather OR wind OR sun OR wind* OR sun* |

While some studies may take measurements on the level of pesticide contamination of the environment, we are only including experiments where a measured impact on bees or bee collected products is recorded. This will have excluded some studies where, for example, a mitigation measure was tested for its impact on pesticide residues in floral pollen and nectar. This is because the matrix has not been utilised by a bee, so it is not a measured effect on bees. As several of the studies demonstrate, pesticide application can produce a repellent effect, and so directly measuring environmental pesticide levels may not accurately reflect the exposure bees face. Additionally, this was chosen to help define the scope and ensure the workload was feasible, and likely to be captured by search terms regarding bees. So, while these studies do meaningfully inform mitigation measures, they are nonetheless outside the scope of this review.

Because we are assessing the efficacy of mitigation measures, this requires there to be an appropriate control in place, which allows for an assessment of the efficacy of the intervention.

Our search terms, and exclusion criteria are neutral to the setting in which the pesticide is used, i.e. agriculture, and forestry, amenity and consumer use, so as to avoid bias towards the use case. The inclusion of some adjuvants with a pesticide application can meaningfully impact their toxicity to bees (Wernecke et al. 2021), so not adding certain adjuvants to certain pesticide applications could be a mitigation measure. However, this topic is reviewed in depth by Straw et al. (2022), and as such has not been covered here.

We chose to use bees as our study taxa rather than pollinators more generally because comprehensively defining a pollinator for the search terms would have drastically increased the volume of publications to screen beyond a workable level. We acknowledge that there may be some evidence for other pollinators, although given bees are commonly used as model species for all pollinators, the scale of this evidence is likely to be small.

While reducing the concentration or rate of application of a pesticide could substantively reduce its toxicity to bees, this was not considered as a mitigation measure within the scope of this study for three reasons. Firstly, it is very common for laboratory studies to test multiple concentrations of a pesticide, with LD50’s for example be conducted for nearly all pesticides. Accordingly, the scale of literature would be impossible to capture meaningfully. Secondly, studies using different concentrations of a pesticide rarely directly relate to a real pesticide exposure scenario, often instead measuring abstract hazard rather than real world risk. The methodological complexity associated with interpreting the concentration/application rate relative to a field realistic spray concentration/application rate is far beyond the scope of this study. Particularly considering that multiple products exist per active ingredient with different rules, and then the additional complexity of different rules between territories and over time. Finally, reducing a pesticides concentration is often ill advised, as it can lead to ineffective control of the pest species facilitating the evolution of resistance (Gressel, 2010). While some studies do directly compare different concentration/application rates in a field realistic setting, they are excluded as a line of evidence to avoid the complexity associated with defining what does and does not constitute applicable evidence, and this is a limitation of our study.

﻿

**Search protocol**

Because many publications were not framed around the idea of mitigation, but nonetheless contained data on the topic, we did not exclude papers based on their title. Instead, abstracts were screened, and those lacking relevance to bees or pesticides being excluded, as well as those not mentioning experimentation related to a mitigation measure.

At all stages, caution was maintained, and ambiguous publications retained rather than excluded to promote comprehensiveness. Publications without abstracts (as complied by Web of Science) were screened by the title initially, with only obviously highly irrelevant titles being excluded, with most being checked in full prior to exclusion. Those not excluded based on their abstracts were read in full and the full exclusion criteria were applied. Studies not accessible online, after searching on Google, Google Scholar and Web of Science were excluded. Abstract searches did not use wildcards before words because this functionality was not supported. All search categories, not just Title, Topic and Abstract were used for supplementary searches due to the lower volume of responses.

Literature on the repellent effect of a pesticide formulation or active ingredient was not included unless it was specifically framed around the idea of that repellence being used to protect bees. This is because many publications were framed around bees avoiding pesticides, usually insecticides, damaging to their health, which is not a mitigation measure a pesticide user can take. It is distinct from the idea of an additional repellent property being added to the application.

**Edge case definitions:**

Some studies tested pesticides for repellence (i.e. Naumann, Currie and Isman, 1994, and Solomon and Hooker, 1989). This is not grounds for inclusion, because they are testing the effects of a pesticide, rather than a mitigation measure which can be applied to a pesticide. The line is blurred in Solomon and Hooker, (1989), which suggests using fungicides as repellents (found to be effective) and ignoring their fungicidal properties. This part of Solomon and Hooker, (1989) has been excluded, but the publication also tests non-pesticidal chemicals, so the publication is included regardless. Naumann, Currie and Isman, (1994) and several other publications looking at the repellence of pesticides were excluded.

In the field of repellence, there is lots of research from pre-2000, much of it from Asia. Many of the articles included in this survey reference or discuss research on repellence not included in this systematic review, however, due to language barriers, poor accessibility to older publications and an apparent lack of online archiving for some of the journals used frequently, many studies which appeared to be relevant could not be found. The discussion on repellence, and critiques of it, mainly centre around overreliance on honeybee research, and none of the unfindable publications appeared to use other taxa. So, while some publications may exist, and be accessible somehow, it is unlikely to change the central conclusions on the topic.

Due to the broad span of fields covered under pesticide mitigation measures, it would not be appropriate to attempt a meta-analysis on the literature captured.

**Assessment of bias**

There is a time lag between publication and indexing on databases which may mean relevant literature published recently was missed, as well as literature published between the time of the search and publication of this article. Literature from before the 1980’s is often not digitised and indexed correctly, so some literature may have been missed due to this. As an English only search, there is a bias which may fail to represent data reported in other languages. As with all research fields there is likely to be a degree of publication bias, but in the absence of a quantitative synthesis this will not have overly impacted our conclusions.

Groups like the International Commission for Plant-Pollinator Relationships and OECD Chemicals committee are key conduits of industry information into government, and these are sources of information not consistently captured in the systematic review because of how they are published. As mitigation measures span a range of fields, and is not a single codified field, it is probable that there is literature not captured by the systematic review, although it is unlikely that the scale of this literature would change any central conclusions of this publication.

**Ambiguous inclusions**

**Alternative forage**

Obuko et al. (2021) had no formal control group, and the methodology was poorly tailored to detect inter-colony visitation. As such it is ambiguous as to whether this methodology qualifies for inclusion in the systematic review, as they did not use a conventional control. It was included as they used inter-colony variation to assess the scale of the effect.

**Medicine**

A host of studies have considered the impacts of nutrition on bees susceptibility to pesticides (e.g. Wintermantel et al. 2022, Linguadoca et al. 2021, Tong, Nieh and Tosi, 2019). They typically find that certain mixes of pollen are effective in reducing bees susceptibility to pesticides. This could be a direct medicinal effect, or a general health boost effect promoting resilience. As such, these are not included within this systematic review, because they’re not interventions with the aim of reducing pesticide harm, but instead illustrate factors that generally boost bee health.

We have drawn the distinction with providing supplemental forage, a mitigation measure we have included. This is because supplemental forage was a workable cut off point, while studying nutrition was infeasible due to the scale and breadth of this literature. Providing supplemental forage is an action a farmer/grower can take, while nutrition is a more ephemeral concept. To actively provide better nutrition, supplemental forage must be supplied, hence it is the supplemental forage, which is the mitigation measure, not the nutrition itself. Inherently as a non-codified field, the distinctions between these topics are a bit blurred. Accordingly, we would encourage those interested in the impacts of supplemental forage and nutrition to continue to develop this field.

**Repellents**

The literature on the concept of substance repellent to bees is complex. Much of it came prior to digitisation of publications and as such is inaccessible. Further, repellence studies have not always been conducted in the context of developing safer pesticides. Instead many publications used a blue skies science approach. Because of this, there are some publications we lacked access too, or could not include because they lacked relevance to pesticides.

Solomon and Hooker (1989) tested a repellent chemical, and contextualised the results around pesticides. Because of this it was included in the formal review. Sánchez et al. (2021) tested repellents as a method to avoid damage to medfly-monitoring traps. There was no contextualisation or mention of using the chemicals within pesticides. Accordingly, this publication was excluded for being off topic. An entire review could be dedicated to understanding how repellent chemicals could be applied to pesticide applications, and in a more targeted review this borderline literature should be considered.

**Crop stage**

While only one scientific publication on this topic has been identified, the bulk of evidence of this measure likely comes from the residue trials required as part of the submission process for pesticides. Both the European Food Safety Authority and the American Environmental Protection Agency require data on the residue levels of pesticides after application. Different studies apply the pesticide at different crop stages, so while no regulatory study is known to the authors directly comparing application at different crop stages, comparison between trials would yield data on the efficacy of this measure.

**Irrigation**

There is evidence of reduced pesticide residues in floral nectar and pollen with irrigation (McCurdy et al. 2017), although this is not included as it is not a bee collected matrix.

**Section S2: Definitions**

Publications can span topics if they include multiple experiments or have a methodology which ambiguously fits multiple definitions.

Modelling- Publications which make mathematical predictions parametrised to bees, but do not actually collect data using bees.

Lab- Any study conducted indoors, or with bees not free flying outdoors.

Semi-field- Any study in an exclusion cage. Must use free flying bees. Still using a methodology to replicate the mitigation measure, rather than directly applying it in the field.

Field- A direct application of the mitigation measure in an outdoor setting.

**Identification of studies via databases and registers**

Records removed *before screening*:

Duplicate records removed (n = 1390)

Records identified from Web of Science:

Databases (n = 4427)

Records identified from literature

Databases (n = 1)

**Identification**

Records screened-Abstract

(n = 3037)

Records excluded**

(n = 2639)

**Screening**

Reports sought for retrieval

(n = 352)

Reports not retrieved

(n = 16)

Reports excluded:

Ineligible (n = 302)

Reports assessed for eligibility

(n = 336)

**Included**

Studies included in review

(n = 34)

**Figure S1.** A PRISMA flow diagram detailing the exclusion of publications at various stages (Page et al. 2020).

| Search Term | Results Returned |
| --- | --- |
| (Dropleg) | 2 |
| (Alternative Forage OR Alternative Resources) | 43 |
| (Waterer OR Water Feeder) | 4 |
| (Irrigation OR Sub-Irrigation) | 28 |
| (Repellent) | 64 |
| (Dusk OR (Crop Stage) OR Evening OR Night OR Time of day OR Hour OR BBCH* OR Month OR Season) | 701 |
| (Covering) | 134 |
| (Nozzle OR Droplet OR Engineering Controls) | 125 |
| (Buffer Zone OR Buffer Strip OR No Spray Strip OR No-Spray Strip) | 16 |
| (Spray Height OR Boom Height) | 3 |
| (Formulation Type) | 13 |
| (Flowering Weeds) | 33 |
| (Aerial) | 33 |
| (Dust) | 78 |
| (Water Volume OR Application Volume) | 25 |
| (Mow OR Mowing) | 18 |

**Supplementary Table 2.** The number of results returned from the additional low yielding searches, including the terms used. The terms in the Search Term column accompany the bee term ((honeybee OR bumblebee OR bee OR bees) and the pesticide term ((pesticid* OR herbicid* OR fungicid* OR adjuvant OR co-formulant OR coformulant OR insecticide OR molluscicide OR (plant protection product) OR agrochem* OR (agro-chem*) OR agrichem* OR (agri-chem*))), each linked with the AND function.

**Figure S2**. A heat map showing where the publications originated from.

**References**

Gressel, J. (2010) Low pesticide rates may hasten the evolution of resistance by increasing mutation frequencies. *Pest Management Science*, **67**, 253-257.

Sánchez, D., Hernández, F., Sánchez, P., and Gómez, E. (2021). When friends become unfriendly: testing repellents against a stingless bee (*Trigona corvina* Cockerell) that damages medfly‐monitoring traps. *International Journal of Tropical Insect Science*, **42**, 977-982.

Tong, L., Nieh, J.C., and Tosi, S. (2019). Combined nutritional stress and a new systemic pesticide (flupyradifurone, Sivanto®) reduce bee survival, food consumption, flight success, and thermoregulation. *Chemosphere*, **237**, 124408.

Naumann, K., Currie, R.W., and Isman, M.B. (1994) Evaluation of the repellent effects of a neem insecticide on foraging honey bees and other pollinators. *The Canadian Entomologist*, **126**, 225-230.

﻿McCurdy, J.D., Held, D.W., Gunn, J.M., and Barickman, T.C. (2017) Dew from warm-season turfgrasses as a possible route for pollinator exposure to lawn- applied imidacloprid. *Crop, Forage & Turfgrass Management*, **3**, 1-6.

Linguadoca, A., Rizzi C., Villa, S., and Brown, M.J.F. (2021). ﻿Sulfoxaflor and nutritional deficiency synergistically reduce survival and fecundity in bumblebees. *Science of the Total Environment*, **795**, 148680.

Page, M.J., McKenzie, J.E., Bossuyt, P.M., Boutron, I., Hoffmann, T.C., Mulrow, C.D., et al. (2020) The PRISMA 2020 statement: an updated guideline for reporting systematic reviews. *British Medical Journal*, **372**, n71.

Wernecke, A., Eckert, J.H., Forster, R., Kurlemann, N., and Odemer, R. (2021) Inert agricultural spray adjuvants may increase the adverse effects of selected insecticides on honey bees (*Apis mellifera* L.) under laboratory conditions. *Journal of Plant Disease Protection*, **129**, 94-105.

Wintermantel, D., Pereira-Peixoto, M-H., Warth, N., Melcher, K., Faller, M., Feurer, J., Allan, M.J., Dean, R., Tamburini, G., Knauer, A.C., Schwarz, J.M., Albrecht, M., and Klein, A.M. (2022) Flowering resources modulate the sensitivity of bumblebees to a common fungicide. *Science of the Total Environment*, **829**, 154450.
